# Decreasing the Non-Fermentable Sugar Concentration in *Aspergillus niger* by Destroying the α-Glucosidase Genes

**DOI:** 10.3390/jof12100764

**Published:** 2026-10-11

**Authors:** Zhengyu Liu, Shiwei Mi, Dandan Li, Kexin Zhao, Di Ma, Yuhan Jiao, Fuping Lu, Ming Li

**Affiliations:** 1 State Key Laboratory of Bio-Based Fiber Materials, Tianjin University of Science and Technology, Tianjin 300457, China; liuzhengyu397@163.com (Z.L.); mswdyx163@163.com (S.M.); lidd1210@163.com (D.L.); zkxc0327@163.com (K.Z.); dm13835707932@163.com (D.M.); 16629067189@163.com (Y.J.); 2 Key Laboratory of Industrial Fermentation Microbiology, Ministry of Education, Tianjin University of Science and Technology, Tianjin 300457, China; 3 College of Biotechnology, Tianjin University of Science and Technology, Tianjin 300457, China

**Keywords:** non-fermentable sugar, *Aspergillus niger*, CRISPR-Cas9, α-glucosidase

## Abstract

*Aspergillus niger* is the main microbial cell factory for industrial citric acid production, but 2–3% of residual sugar typically remains unutilized in the fermentation broth. To reduce carbon loss caused by residual sugars, seven α-glucosidase genes potentially involved in non-fermentable sugar formation were investigated. In this study, seven α-glucosidase gene-disrupted strains were constructed and were designated as An214233, An119858, An52452, An50927, An50055, An49940, and An40261. Disruption of each gene reduced isomaltose accumulation and increased citric acid production; at the same time, some morphological characteristics of the strain also changed. Among them, the isomaltose content in the fermentation broth of An214233, An50927, An50055, and An40261 decreased by more than 30%, indicating that these genes play important roles in isomaltose formation. However, the reduction in isomaltose content was not directly proportional to the increase in citric acid production. An49940 exhibited the greatest increase in citric acid production despite a relatively moderate reduction of 13.62% in isomaltose content. This strain also exhibited favorable overall performance in total and reducing sugar utilization, together with smaller mycelial pellets and thicker hyphae. These results indicate that citric acid production is influenced not only by non-fermentable sugar formation but also by sugar utilization and mycelial morphology, thereby providing a theoretical basis and potential genetic targets for reducing carbon source loss, improving citric acid production, and decreasing residual sugars in industrial fermentation.

## 1. Introduction

Citric acid (CA) is an important organic acid, appearing as colorless crystals that often contain one molecule of water of crystallization. It is odorless, has a strong acidic taste, is easily soluble in water, and is widely used in food, medicine, daily chemical and other industries [1,2]. In 2025, the global citric acid market volume reached approximately 2.93 × 10^6^ t [3], while citric acid production in China reached approximately 2.18 × 10^6^ t [4]. The global citric acid market is projected to reach USD 5.10 billion by 2035 [5].

**Figure 1:**
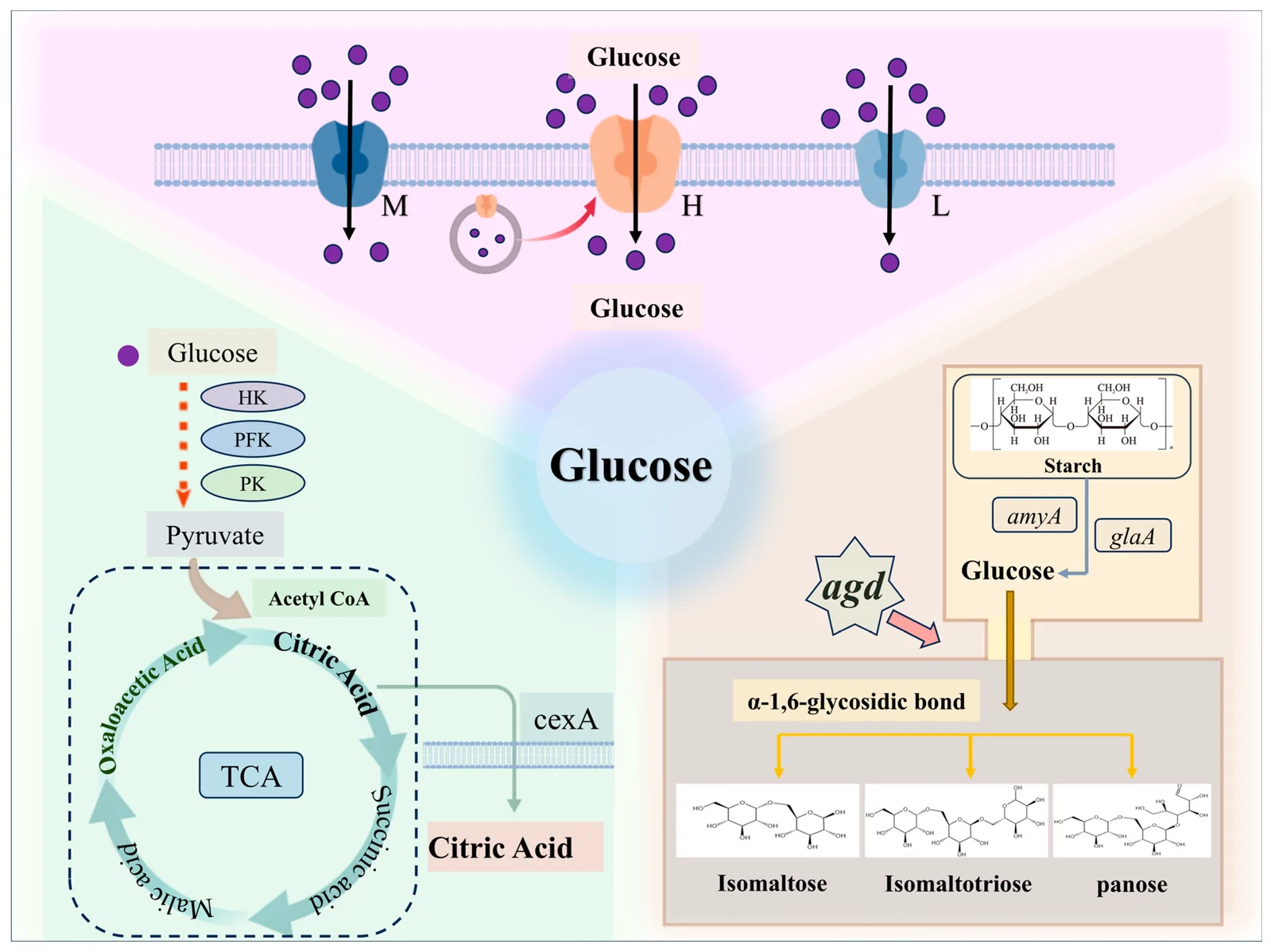
Schematic diagram of glucose transport, non-fermentable sugar formation, and the citric acid synthesis pathway in *A. niger*. Purple circles represent glucose. Arrows indicate the direction of glucose transport, metabolic conversion, citrate export, and transglycosylation. M, medium-affinity glucose transporter; H, high-affinity glucose transporter; L, low-affinity glucose transporter. HK, hexokinase; PFK, phosphofructokinase; PK, pyruvate kinase. TCA, tricarboxylic acid cycle; *cexA*, citrate exporter; *amyA*, amylase; *glaA*, glucoamylase; *agd*, α-glucosidase encoding gene.

Currently, citric acid is produced industrially by submerged fermentation, in which the Simultaneous Saccharification and Fermentation method is commonly employed [6]. During this process, the initial sugar concentration for citric acid production by *A. niger* ranges from 100 to 180 g·L^−1^ [7,8]. The theoretical yield of citric acid is 1.067 g/g glucose. However, the actual sugar-to-acid conversion rate in industrial production is only approximately 95% [9]. This gap between theoretical and actual yields can be primarily attributed to the fact that 2–3% of the sugar remains unutilized at the end of fermentation [10]. To facilitate the understanding of the molecular basis of residual sugar formation, Figure 1 shows the metabolic pathway of glucose transport, utilization, and transglycosylation leading to non-fermentable sugars in *A. niger*.

To reduce residual sugar content during citric acid fermentation, researchers have adopted various measures. Wang et al. [11] used the strategy of adding saccharification enzymes in stages during fermentation. The citric acid titer was increased by about 13.3%, but the residual sugar content was still high. Xue et al. overexpressed a high-affinity glucose transporter protein, HGT1, in *A. niger* CGMCC 10142 [12]. Although the residual sugar content in the fermentation broth was reduced to some extent, a residual sugar level of 10.6 g/L was still detected at the end of fermentation.

Sugar can not only enter cells and be utilized but also undergo transglycosylation to form non-fermentable sugars, and the production of non-fermentable sugars is associated with α-glucosidase [13]. To date, only a few of these genes have been functionally characterized. For instance, in 2016, Wang et al. [14] knocked out the *agdA* (214233) gene in a citric acid-producing strain, resulting in a 62.5% reduction in α-glucosidase activity and rendering isomaltose almost undetectable in the fermentation broth, indicating that *agdA* is a major contributor to transglycosylation. However, residual isomaltose was still detected under certain conditions, suggesting the involvement of other α-glucosidase genes. In 2019, Ma et al. [15] found that another α-glucosidase gene (*agdB* (119858)) can also catalyze transglycosylation reactions. Despite these advances, a comprehensive understanding of the transglycosylation process in *A. niger* requires systematic characterization of all α-glucosidase genes, as their differential contributions to non-fermentable sugar formation and their potential impacts on citric acid production have not been fully elucidated.

α-Glucosidase [16] (EC 3.2.1.20) is an exoglycosidase that hydrolyzes α-1,4-glycosidic bonds and catalyzes transglycosylation at the nonreducing end of oligosaccharides to form α-1,6-glycosidic bonds, generating non-fermentable sugars such as isomaltose, isomaltotriose, and panose [17,18]. In 2025, Tissopi et al. [19] reported that the *agdB* of *A. neoniger* has strong transglycosylation activity. Yuan et al. [20] found that in *A. niger* CBS 513.88, there are seven genes associated with α-glucosidase activity. However, in *A. niger* ATCC 1015, functional characterization has been reported only for *agdA* and *agdB*, while the roles of the other five genes in transglycosylation and non-fermentable sugar formation remain unexplored.

In this study, using AnYX0607 as the starting strain, we individually knocked out seven α-glucosidase genes to investigate their effects on colony morphology, mycelia, and mycelial pellets, their contributions to transglycosylation activity, and their impacts on citric acid production. This study provides new insights into the genetic basis of transglycosylation activity in citric acid-producing *A. niger* strains and has implications for the construction of engineered strains with efficient sugar utilization and reduced residual sugar.

## 2. Materials and Methods

### 2.1. Strains, Plasmids, Primers, Media, and Cultivation

The strains and plasmids used in this study are listed in Table S1. The primers in this study are listed in Table S2. The industrial citric acid-producing strain AnYX0607 was used as the parental strain. *Escherichia coli* JM109 was used as the host for the propagation of plasmid DNA. The bacterial strains were cultivated in LB medium at 37 °C. Potato dextrose agar (PDA) was used for the growth of *A. niger*. The *A. niger* liquid medium contained 20 g·L^−1^ anhydrous glucose (Tianjin Kermel Chemical Reagent Co., Ltd., Tianjin, China); 10 g·L^−1^ yeast extract (Oxoid Ltd., Basingstoke, UK); 1 g·L^−1^ MgSO_4_·7H_2_O (Shanghai Macklin Biochemical Co., Ltd., Shanghai, China); and 1 g·L^−1^ K_2_HPO_4_ (Shanghai Macklin Biochemical Co., Ltd., Shanghai, China); the pH was adjusted to 4. Crushed corn was liquefied with amylase to obtain the liquefied corn medium. A total of 1 × 10^5^ spores mL^−1^ was inoculated into the liquefied corn medium and cultivated at 220 r·min^−1^ and 35 °C for 96 h.

### 2.2. PCR Amplification and Sequencing of α-Glucosidase Genes

In this study, using the genome of *A. niger* strain AnYX0607 as a template, the gene sequences of seven α-glucosidases were obtained by PCR with the primers listed in Table S2. The PCR products were sent to Genewiz (South Plainfield, NJ, USA) for sequencing, and the resulting sequences were compared with the corresponding sequences from *A. niger* ATCC 1015 using NCBI BLAST(https://blast.ncbi.nlm.nih.gov/Blast.cgi; website accessed on 18 December 2024).

### 2.3. Determination of Residual Sugars in Industrial Fermentation Broth by GC–MS

The fermentation broth of the original strain AnYX0607 was centrifuged, and the residual sugars in the supernatant were analyzed by GC-MS [21]. The fermentation supernatant was derivatized with a silylation reagent (Merck KGaA, Darmstadt, Germany). GC-MS analysis was performed on a Shimadzu GCMS-QP2010 instrument (Shimadzu Corporation, Kyoto, Japan) equipped with an Rtx-5MS capillary column (30 m × 0.25 mm × 0.25 μm; Restek Corporation, Bellefonte, PA, USA). High-purity helium was used as the carrier gas at a constant flow rate of 1.00 mL/min. The injector temperature was set at 250 °C, and 1.0 μL of sample was injected in split mode (split ratio 20:1). The oven temperature program was as follows: initial temperature held at 100 °C for 3 min, then increased to 250 °C at a rate of 10 °C/min and maintained for 30 min. The MS interface temperature was 250 °C, and the ion source temperature was 230 °C. Mass spectrometric detection was performed in electron ionization (EI) mode at 70 eV. Compounds were identified by comparison of retention times and mass spectra with authentic standards and the NIST library.

### 2.4. Construction of the α-Glucosidase Gene Disruption Plasmid

**Figure 2:**
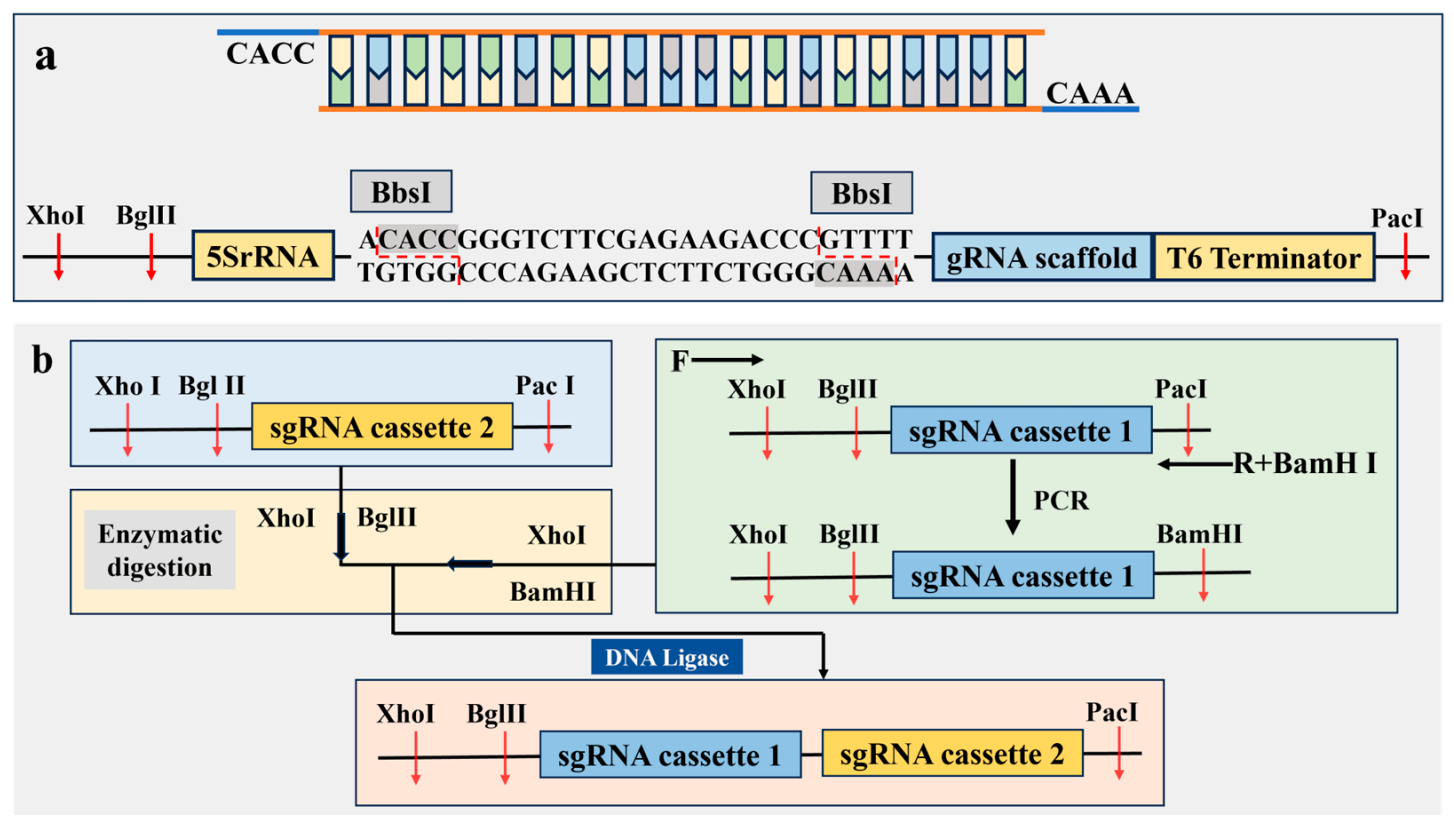
Schematic diagram of dual sgRNA expression cassette construction: (**a**) schematic diagram of single-target sgRNA expression plasmid construction, showing the 5S rRNA promoter, target sequence, gRNA scaffold, and T6 terminator; (**b**) schematic diagram of tandem sgRNA expression cassettes. Different colors distinguish the functional elements and sgRNA cassettes. F and R indicate the forward and reverse primers, respectively. The labels XhoI, BglII, BbsI, BamHI, and PacI indicate the corresponding restriction enzyme sites. Red arrows indicate restriction enzyme cleavage sites, black arrows indicate the direction of PCR amplification or fragment assembly, and dashed lines indicate the cohesive (sticky) ends generated by restriction enzyme digestion.

To improve the efficiency of precise genome editing, we used CRISPR-Cas9 technology and designed two sgRNAs to construct an α-glucosidase gene disruption plasmid. A 20-bp target sequence for each α-glucosidase gene was designed using CRISPOR (http://crispor.tefor.net; accessed on 17 February 2025) [22]. The designed α-glucosidase gene target sequences were synthesized by Genewiz (South Plainfield, NJ, USA). A schematic diagram of the construction of the dual-sgRNA expression plasmid is shown in Figure 2.

Each of the double-sgRNA intermediate expression plasmids targeting distinct α-glucosidase genes was digested with BglII and PacI. The purified DNA fragments were then ligated into the pFC332 vector that had been digested with the same restriction enzymes, yielding the pFC332-target-sgRNA disruption plasmid, each containing two tandem sgRNA expression cassettes.

### 2.5. Construction of a Donor DNA Fragment Containing the glaA Expression Cassette

**Figure 3:**
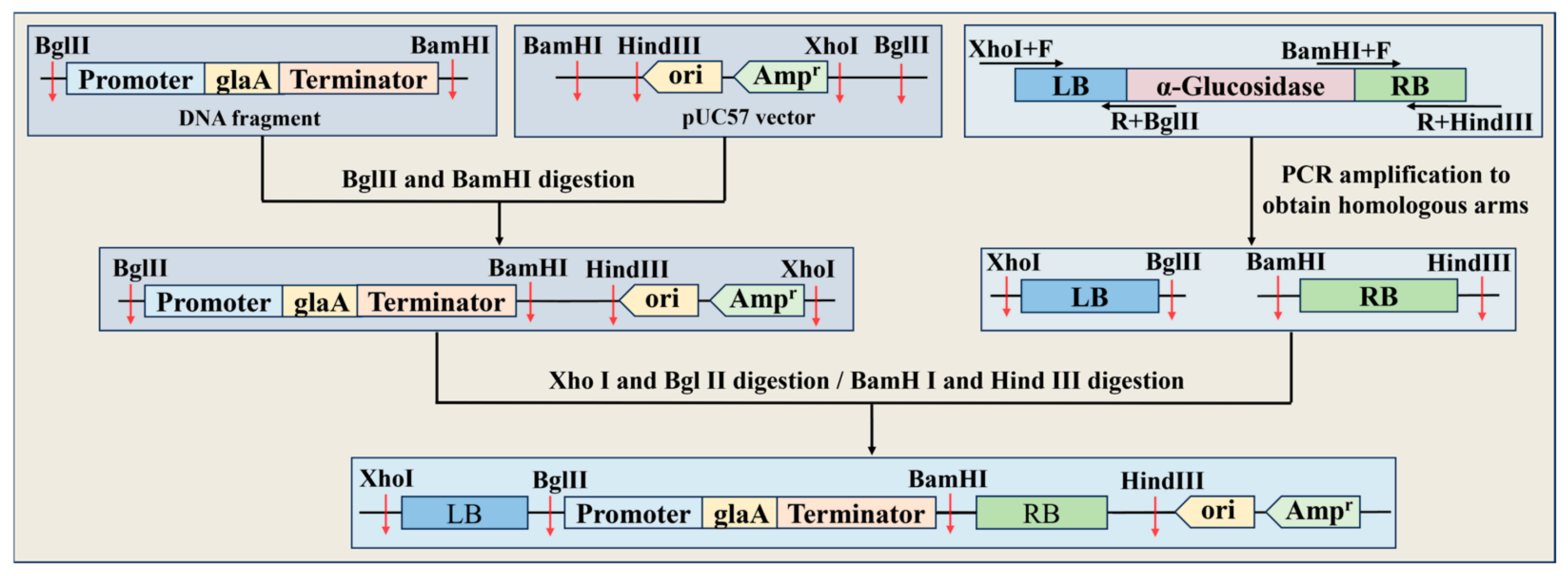
Schematic diagram of the donor fragment containing upstream and downstream homologous arms and the *glaA* expression cassette. Different colors distinguish the functional elements. F and R indicate the forward and reverse primers, respectively; LB and RB indicate the left and right homologous arms; ori indicates the origin of replication; and Amp^r^ indicates the ampicillin-resistance gene. Red arrows indicate restriction enzyme cleavage sites, and black arrows indicate the workflow of digestion, PCR amplification, and DNA ligation.

The *glaA* expression cassette was constructed by overlap extension PCR. This cassette was then ligated between the BglII and BamHI sites of pUC57 to generate the recombinant plasmid pUC57-glaA. Using the genomic DNA of AnYX0607 as a template, the left (LB) and right (RB) homology arms of the seven α-glucosidase genes were obtained by PCR. The amplified LB was digested with XhoI and BglII and ligated into the pUC57-glaA plasmid to construct the intermediate plasmid pUC57-LB-glaA. Subsequently, the RB was digested with BamHI and HindIII and ligated into pUC57-LB-glaA to obtain the final donor plasmid pUC57-LB-glaA-RB. The seven recombinant plasmids were designated as pUC57-214233-LB-glaA-214233-RB, pUC57-119858-LB-glaA-119858-RB, pUC57-52452-LB-glaA-52452-RB, pUC57-50927-LB-glaA-50927-RB, pUC57-50055-LB-glaA-50055-RB, pUC57-49940-LB-glaA-49940-RB, and pUC57-40261-LB-glaA-40261-RB. Each plasmid contained the LB and RB of a distinct α-glucosidase gene as well as the *glaA* expression cassette. The plasmid construction strategy is shown in Figure 3.

### 2.6. Construction of α-Glucosidase Gene-Disrupted Strains

**Figure 4:**
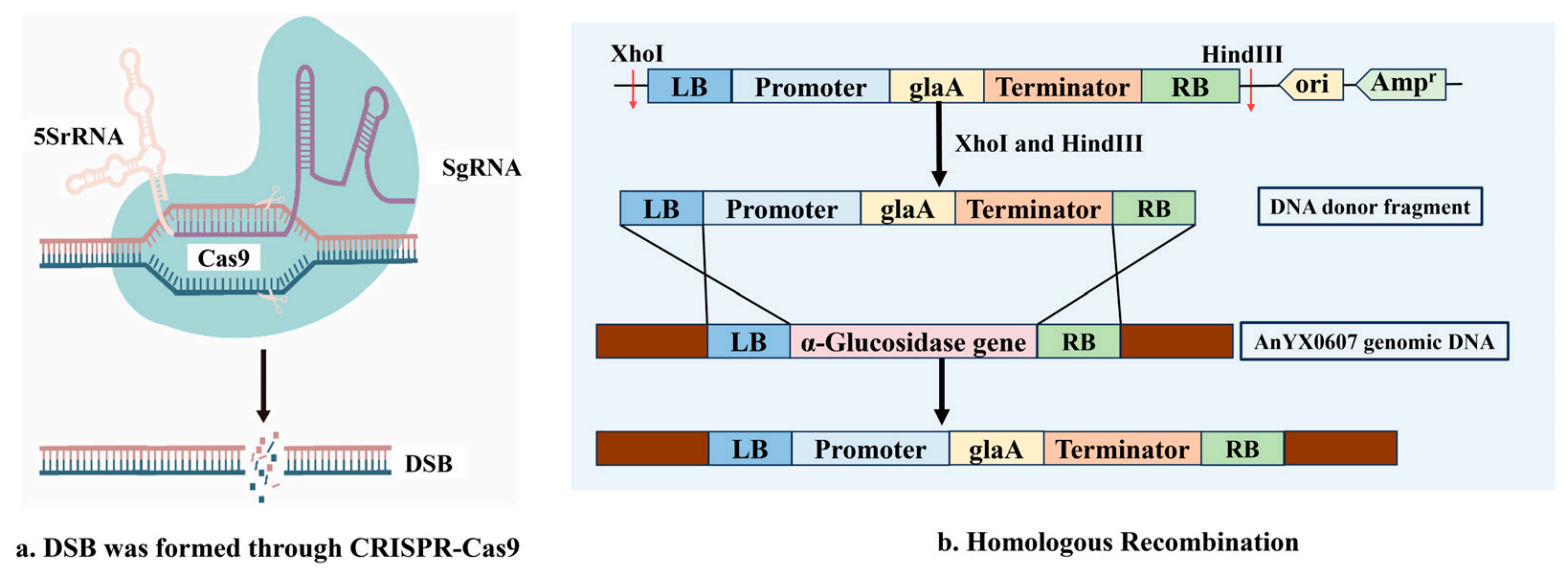
Schematic flowchart of α-glucosidase gene disruption mediated by CRISPR-Cas9 technology: (**a**) DSB was formed through CRISPR-Cas9; (**b**) homologous recombination. Different colors distinguish the genetic elements. LB denotes the upstream homologous arm, RB denotes the downstream homologous arm, ori indicates the origin of replication, and Amp^r^ indicates the ampicillin-resistance gene. Red arrows indicate restriction enzyme cleavage sites, and black arrows indicate the direction of Cas9 cleavage, donor-fragment preparation, and homologous recombination.

To construct the seven α-glucosidase gene-disrupted strains, this study transformed the disruption plasmids and donor fragments into AnYX0607 via PEG-mediated transformation so as to precisely target the corresponding sites in the α-glucosidase genes and achieve gene disruption [23]. The genetic engineering strategy for constructing these gene-disrupted strains is illustrated in Figure 4.

### 2.7. Validation of α-Glucosidase Gene-Disrupted Strains

The constructed transformants were transferred to fresh PDA plates containing hygromycin and cultured for 2 d. Genomic DNA of each transformant was then extracted using the cetyltrimethylammonium bromide (CTAB) method [24] and used as templates for PCR verification.

### 2.8. Biomass Determination

To determine the effect of gene disruption on growth rate, 500 μL aliquots of spore suspensions (10^7^ conidia mL^−1^) of the original strain and each mutant strain were inoculated into *A. niger* liquid medium. The cultures were incubated for 120 h. Every 12 h, samples were taken, and the mycelia were collected by filtration and then dried at 70 °C to constant weight.

### 2.9. Morphological Characterization of α-Glucosidase Gene-Disrupted Strains

To investigate whether the deletion of α-glucosidase genes affects phenotypic characteristics (colony, mycelium, and mycelial pellet), 10 μL aliquots of *A. niger* spore suspensions were inoculated onto PDA medium and cultured for 8 d to observe colony morphology. Aliquots of 500 μL of *A. niger* spore suspensions of both the original strain and the mutants were inoculated onto liquefied corn media for 96 h. Mycelial and mycelial pellets of the original strain and the gene-disrupted strains were observed via a Nikon ECLIPSE Ci (Nikon Corporation, Tokyo, Japan) orthogonal electron microscope with NIS-Elements SF 4.60.00 software (Nikon Corporation, Tokyo, Japan).

### 2.10. Determination of Total Sugar, Reducing Sugar, Isomaltose, and Citric Acid in Fermentation Broth

The reducing sugar content was determined using the 3,5-dinitrosalicylic acid (DNS; Beijing Solarbio Science & Technology Co., Ltd., Beijing, China) method, whereas the total sugar content was determined using the same method following acid hydrolysis of the fermentation broth [25]. The concentrations of isomaltose and CA in the fermentation broth were determined by HPLC using an Agilent 1620 system equipped with a refractive index detector RID G7162A (Agilent Technologies, Inc., Santa Clara, CA, USA) and an Aminex HPX-87H (Bio-Rad Laboratories, Inc., Hercules, CA, USA) column maintained at 55 °C. The mobile phase was 5 mM H_2_SO_4_ (Tianjin Kermel Chemical Reagent Co., Ltd., Tianjin, China) at a flow rate of 0.6 mL min^−1^, and the injection volume was 10 μL.

Reference standards of isomaltose and citric acid were purchased from Shanghai Yuanye Bio-Technology Co., Ltd. (Shanghai, China), with a purity of ≥98% for quantitative analysis. A series of standard working solutions at different concentrations was accurately prepared and analyzed by HPLC. Calibration curves were constructed by plotting the peak areas against the corresponding standard concentrations, and linear regression equations were obtained for quantitative analysis. The linear ranges were 0.1–0.5 mg/mL for isomaltose and 1–5 mg/mL for citric acid.

For each strain, three independent shake-flask cultures were prepared under identical fermentation conditions. Broth from each flask was collected and analyzed separately, and each flask was considered one independent biological replicate. Prior to analysis, fermentation broth samples were pretreated. The original fermentation broths of each strain were diluted 20-fold and filtered through a 0.22 μm aqueous membrane filter (Tianjin Jinteng Experiment Equipment Co., Ltd., Tianjin, China) before injection. The peak area obtained for each individual sample was substituted into the corresponding linear regression equation to calculate the analyte concentration in the diluted sample. The calculated concentrations were then multiplied by the dilution factor of 20 to obtain the concentrations in the original fermentation broths. The values obtained from the three independent shake-flask cultures were retained separately for statistical analysis, with each culture representing one biological replicate.

### 2.11. Statistical Analysis

All statistical calculations were performed using GraphPad Prism version 10 (GraphPad Software, LLC, Boston, MA, USA). One-way ANOVA followed by Dunnett’s multiple-comparison test was adopted to compare each engineered strain with the parental strain AnYX0607. Normality and homogeneity of variance were checked prior to ANOVA. All experiments were performed with three independent biological replicates (*n* = 3). Data are presented as mean ± standard deviation (SD), and error bars indicate SD. Significance levels were defined as ns (*p* ≥ 0.05), * (*p* < 0.05), ** (*p* < 0.01), and *** (*p* < 0.001).

## 3. Results

### 3.1. Analysis of Residual Sugar in Industrial Fermentation Broth

The residual sugar components of the industrial fermentation broth of the original strain AnYX0607 were detected and analyzed by GC-MS. The results (Table 1) showed that isomaltose, a major non-fermentable sugar, exhibited the highest peak-area percentage among the detected saccharides, reaching 69.45%, suggesting that isomaltose was the primary component of the residual sugars. Therefore, the detection of residual sugar components in the fermentation broth in this study was primarily focused on isomaltose.

### 3.2. Comparative Analysis of α-Glucosidase Gene Sequences

In this study, the gene sequences of seven α-glucosidases were obtained via PCR. After sequencing, they were compared using NCBI BLAST, and the results are shown in Table 2. Compared with ATCC 1015, the nucleotide sequence similarities of all α-glucosidase genes in AnYX0607 were higher than 98.86%, and the amino acid similarities exceeded 99.33%. Among them, genes 50927 and 50055 were completely identical (100%) in both nucleotide and amino acid sequences. These results indicate that AnYX0607 shares extremely high homology with ATCC 1015 in terms of α-glucosidase genes.

### 3.3. Construction and Identification of α-Glucosidase Gene-Disrupted Strains

**Figure 5:**
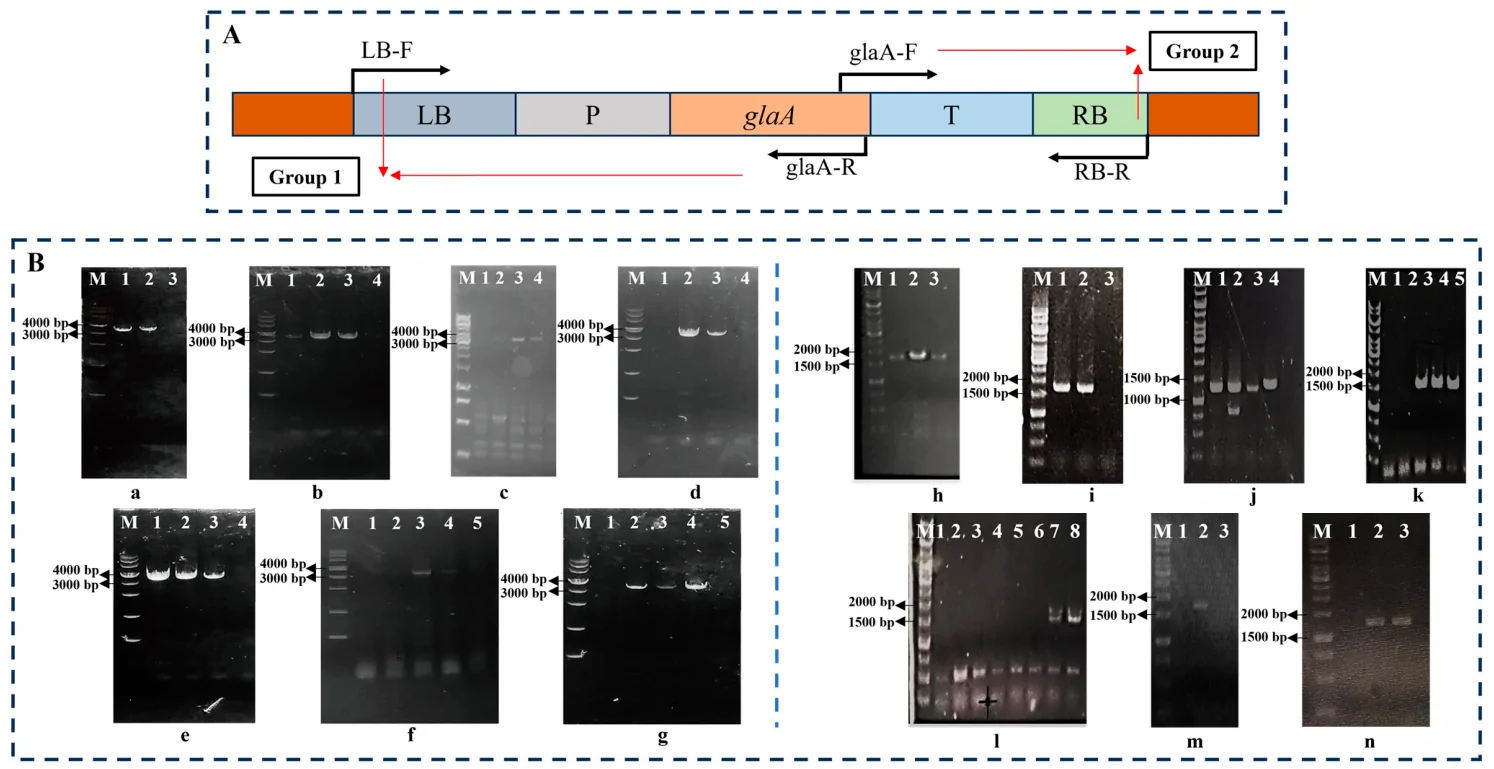
PCR verification of α-glucosidase gene-disrupted strains:(**A**) sites of two independent primer pairs for PCR verification of transformants. Group 1 (LB-F/glaA-R) and Group 2 (glaA-F/RB-R) indicate the two primer pairs, respectively. LB and RB denote the upstream and downstream homologous arms, respectively; P denotes the promoter; *glaA* denotes the inserted gene; and T denotes the terminator. Different colors distinguish the genetic elements. Black arrows indicate the primer orientations, whereas red arrows indicate the pairing relationships between the primers in each group. (**B**) gel images of PCR fragments of the transformants: (**a**,**h**) An214233; (**b**,**i**) An119858; (**c**,**j**) An52452; (**d**,**k**) An50927; (**e**,**l**) An50055; (**f**,**m**) An49940; (**g**,**n**) An40261.

For each of the seven α-glucosidase genes, a corresponding disruption plasmid and donor DNA fragment were constructed and co-transformed separately into the parental strain AnYX0607 using PEG-mediated protoplast transformation. Transformants were verified using two primer pairs: LB-F (primer for the upstream homologous arm) paired with glaA-R (primer downstream of the *glaA* expression cassette), and glaA-F (primer downstream of the *glaA* expression cassette) paired with RB-R (primer for the downstream homologous arm) (Figure 5A). PCR results confirmed the successful construction of seven engineered strains with targeted disruption of the α-glucosidase genes: An214233, An119858, An52452, An50927, An50055, An49940, and An40261. The results are shown in Figure 5B(a–n).

### 3.4. Comparison of Growth Rates Between Gene-Disrupted Strains and AnYX0607

To determine whether the disruption of the α-glucosidase genes affected cell growth, the biomass was assessed by measuring the dry cell weight (DCW), which was normalized per unit culture volume to enable quantitative comparison of growth rates.

**Figure 6:**
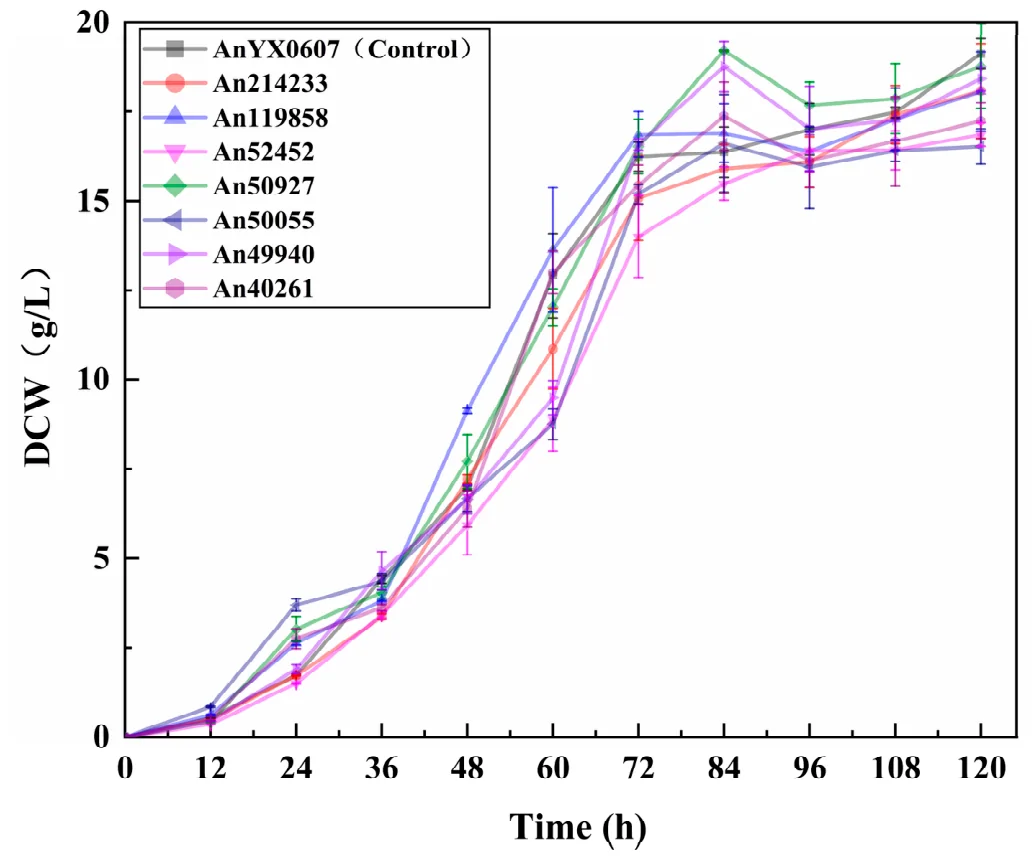
Growth curve comparison between AnYX0607 and gene-disrupted strains.

As shown in Figure 6, the biomass accumulation during fermentation remained largely consistent after α-glucosidase gene disruption. Notably, all strains—including the parental strain AnYX0607 and gene-disrupted mutants An214233, An119858, An52452, An50927, An50055, An49940, and An40261—exhibited identical growth trends throughout the cultivation period. To evaluate differences in final biomass, DCW values at 120 h were statistically analyzed following the method described in Section 2.11. Compared with AnYX0607, strains An52452 and An50055 exhibited significantly lower DCW values, whereas no significant differences in DCW were observed for the other engineered strains. Overall, biomass accumulation remained broadly comparable among the parental and engineered strains.

### 3.5. Comparison of Colony Between Gene-Disrupted Strains and AnYX0607

**Figure 7:**
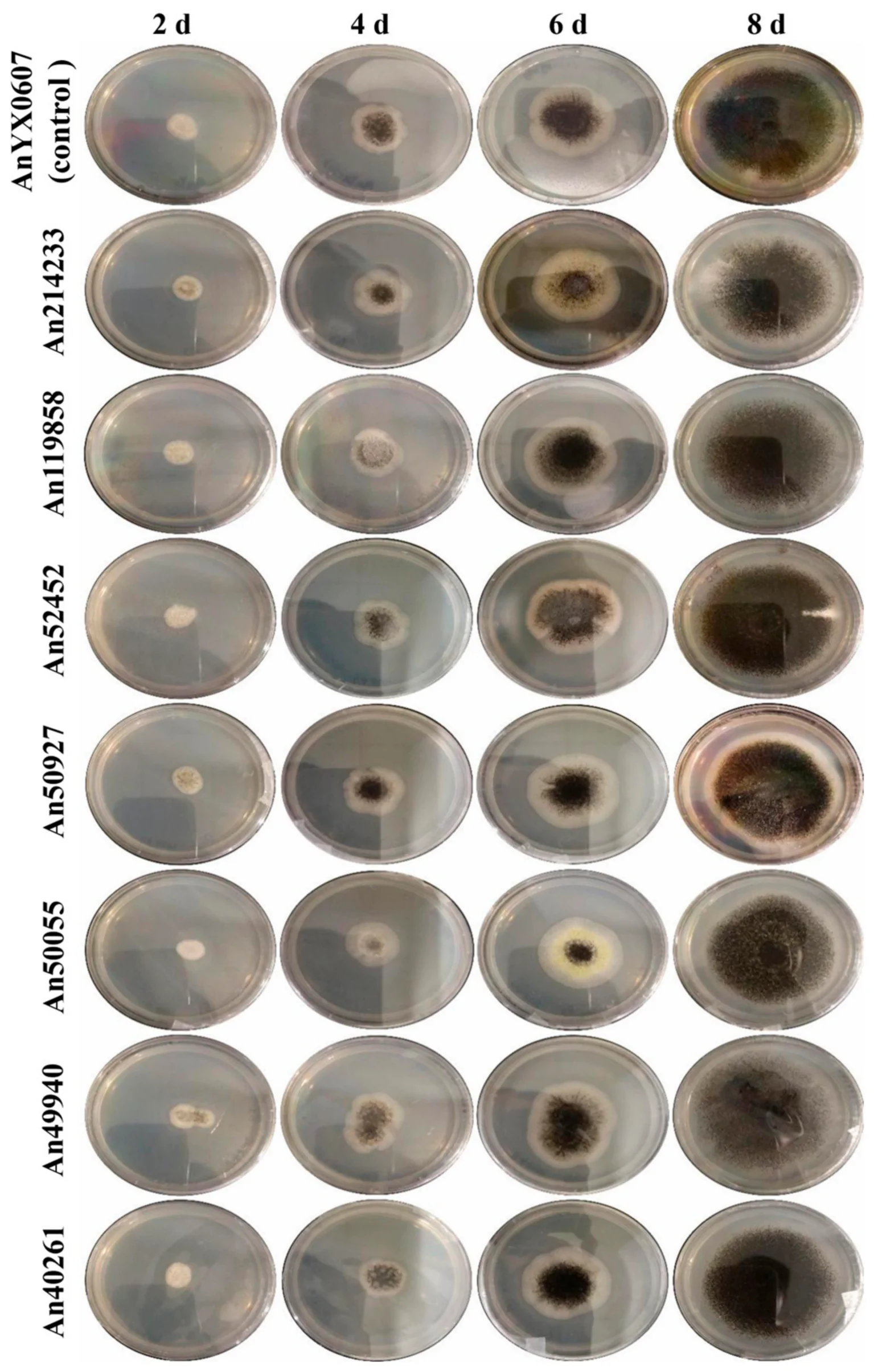
The colony morphology of AnYX0607 and α-glucosidase gene-disrupted strains.

To investigate the effect of α-glucosidase gene disruption on colony growth and morphology, equal amounts of spores from AnYX0607 and the gene-disrupted strains were separately inoculated onto PDA plates and cultured for 8 d, with photographs taken every 48 h during this period. Both the gene-disrupted strains and AnYX0607 exhibited typical fluffy mycelial colony morphology during the initial cultivation period (1–3 d). After 6 d of cultivation, An50055 showed pale yellow peripheral mycelia, indicating incomplete spore maturation and a prolonged spore formation period. After 8 d of growth, all *A. niger* strains formed large, jet-black colonies (Figure 7).

The colony diameters of AnYX0607 and the gene-disrupted strains after 8 d of culture were quantitatively analyzed using the Digital Micrograph imaging system (version 3.6).

**Figure 8:**
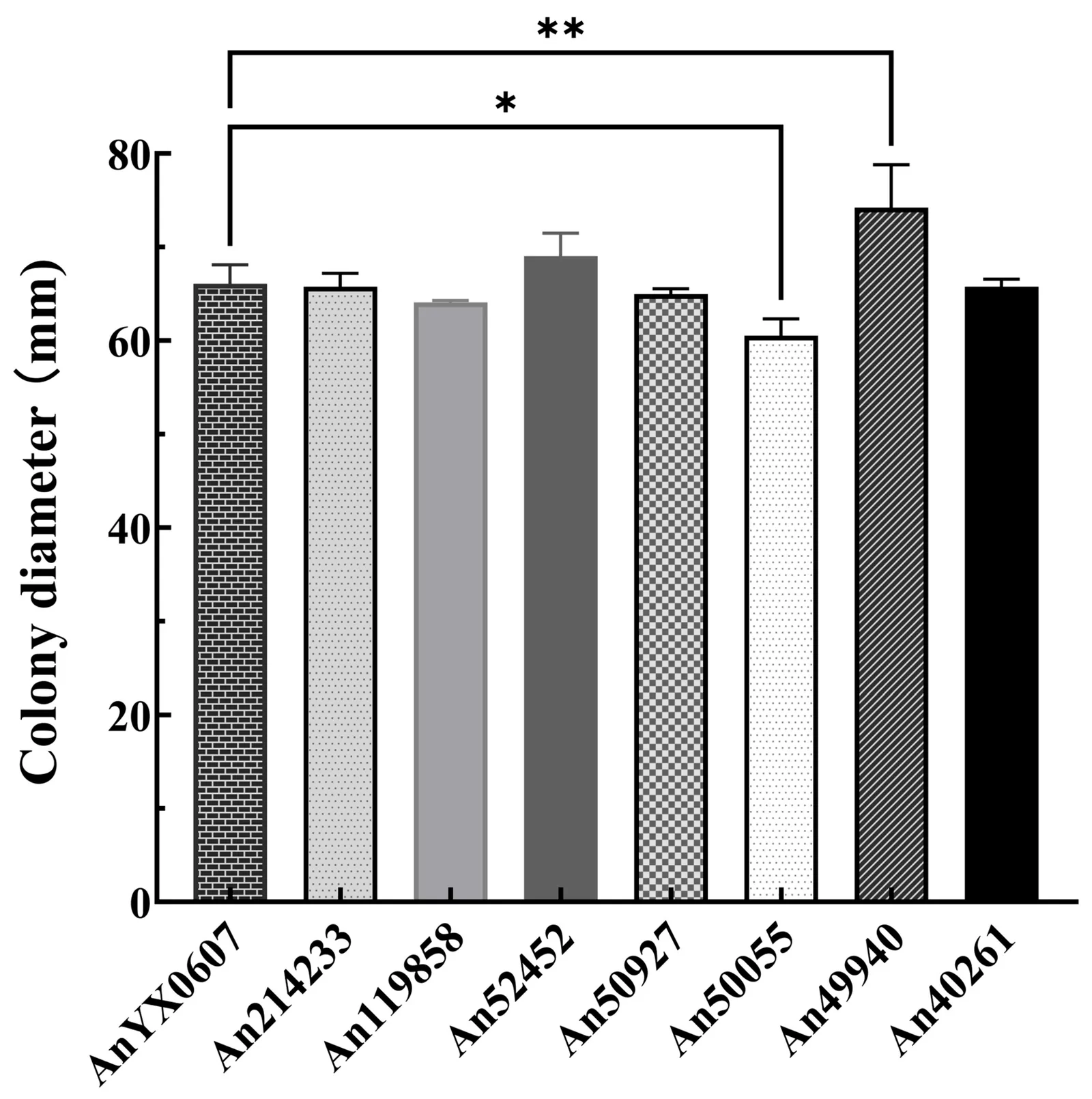
Comparison of plate colony diameters between the α-glucosidase gene-disrupted strains and AnYX0607 (8 d). Data are mean ± SD; *n* = 3; the * symbol represents *p* < 0.05; ** represents *p* < 0.01 compared with AnYX0607.

As shown in Figure 8, after the disruption of α-glucosidase genes, the colony diameters of the strains were generally similar. However, strain An49940 exhibited a significantly larger colony diameter, 12.35% larger than that of AnYX0607, whereas strain An50055 showed a significantly smaller colony diameter, 8.40% smaller than that of AnYX0607.

These results indicate that disruption of different α-glucosidase genes exerts distinct effects on colony growth and sporulation in *A. niger*. The strain An50055 displayed delayed spore maturation, accompanied by a significantly smaller colony diameter relative to the parental strain. In contrast, strain An49940 possessed a markedly larger colony diameter than the parental strain. This suggests functional divergence among α-glucosidase genes in regulating vegetative growth and reproductive development.

### 3.6. Comparison of Mycelial Pellet and Hyphae Diameter Morphology Between Gene-Disrupted Strains and AnYX0607

**Figure 9:**
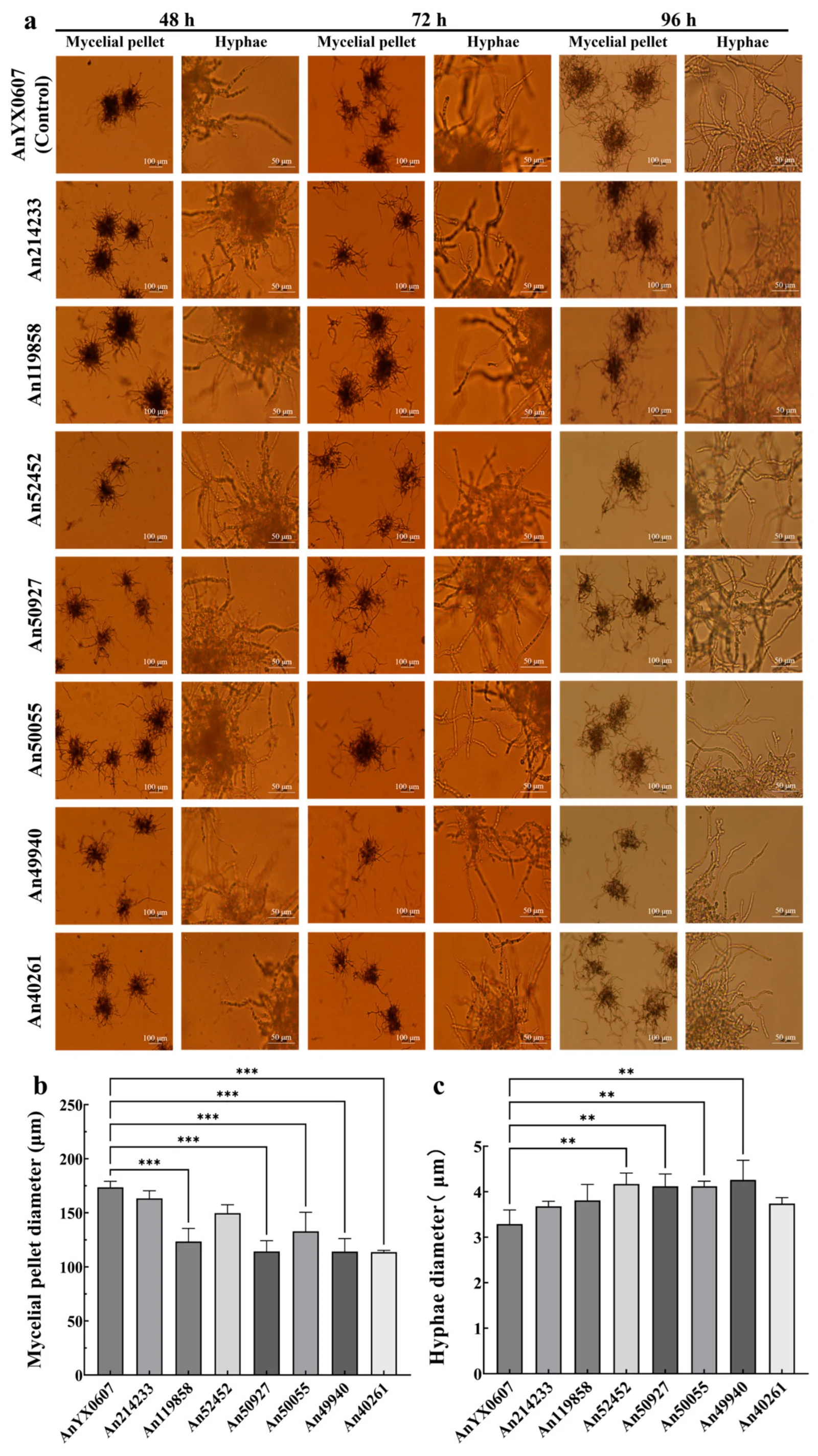
Mycelial pellet and hyphal phenotypes and comparisons of mycelial pellet and hyphal diameters in AnYX0607 and the α-glucosidase gene-disrupted strains: (**a**) Mycelial pellet and hyphal phenotypes. Scale bars: 100 μm for the mycelial pellet micrographs and 50 μm for the hyphal micrographs. (**b**) Comparison of mycelial pellet diameter at 96 h. (**c**) Comparison of hyphal diameter at 96 h. Data in (**b**,**c**) are presented as mean ± SD (*n* = 3); the ** symbol represents *p* < 0.01; and the *** symbol represents *p* < 0.001 compared with AnYX0607.

Morphological observations of mycelial pellets and hyphae of the α-glucosidase gene-disrupted strains and AnYX0607 were performed (Figure 9a). Observations revealed that after 48 h of shake-flask culture, all strains formed white, irregularly spherical or ellipsoidal mycelial pellets, with individual hyphae appearing hyaline and septate. After 72 h of culture, the mycelial pellets became larger and hyphal branching increased. At 96 h, it was observed that the mycelial pellets of AnYX0607 were larger than those of the α-glucosidase gene-disrupted strains, with more fluffy, flocculent hyphae present on the outer layer of the pellets.

Quantitative analysis of the mycelial pellet diameter and hyphal diameter of *A. niger* after 96 h of fermentation was performed using the Digital Micrograph imaging system. The measured mycelial pellet diameters of each strain are shown in Figure 9b, and the measured hyphal diameters are shown in Figure 9c.

As shown in Figure 9b, after 96 h of cultivation, the mycelial pellet diameters of all α-glucosidase gene-disrupted strains were smaller than AnYX0607. The diameters of An119858, An50927, An50055, An49940, and An40261 were significantly reduced, decreasing by 28.83%, 34.20%, 23.45%, 34.24%, and 34.53%, respectively. By contrast, the diameter reductions of An214233 and An52452 were only 5.92% and 13.78%, respectively.

As shown in Figure 9c, after 96 h of cultivation, the hyphal diameters of all α-glucosidase gene-disrupted strains increased compared with AnYX0607. The hyphal diameters of strains An214233, An119858, An52452, An50927, An50055, An49940, and An40261 increased by 11.85%, 15.81%, 26.75%, 25.23%, 25.23%, 29.48%, and 13.68%, respectively. Among these, the hyphal diameters of An52452, An50927, An50055, and An49940 were significantly increased.

In summary, after 96 h of cultivation, all α-glucosidase gene-disrupted strains exhibited smaller mycelial pellets and thicker hyphae. Previous studies have reported that smaller pellets facilitate nutrient and oxygen transfer during fermentation, while thicker hyphae may enhance metabolic activity and product accumulation [26]. Such morphological changes may thereby promote efficient synthesis and accumulation of citric acid in submerged liquid fermentation.

### 3.7. Determination of Isomaltose Content in Gene-Disrupted Strains and AnYX0607

**Figure 10:**
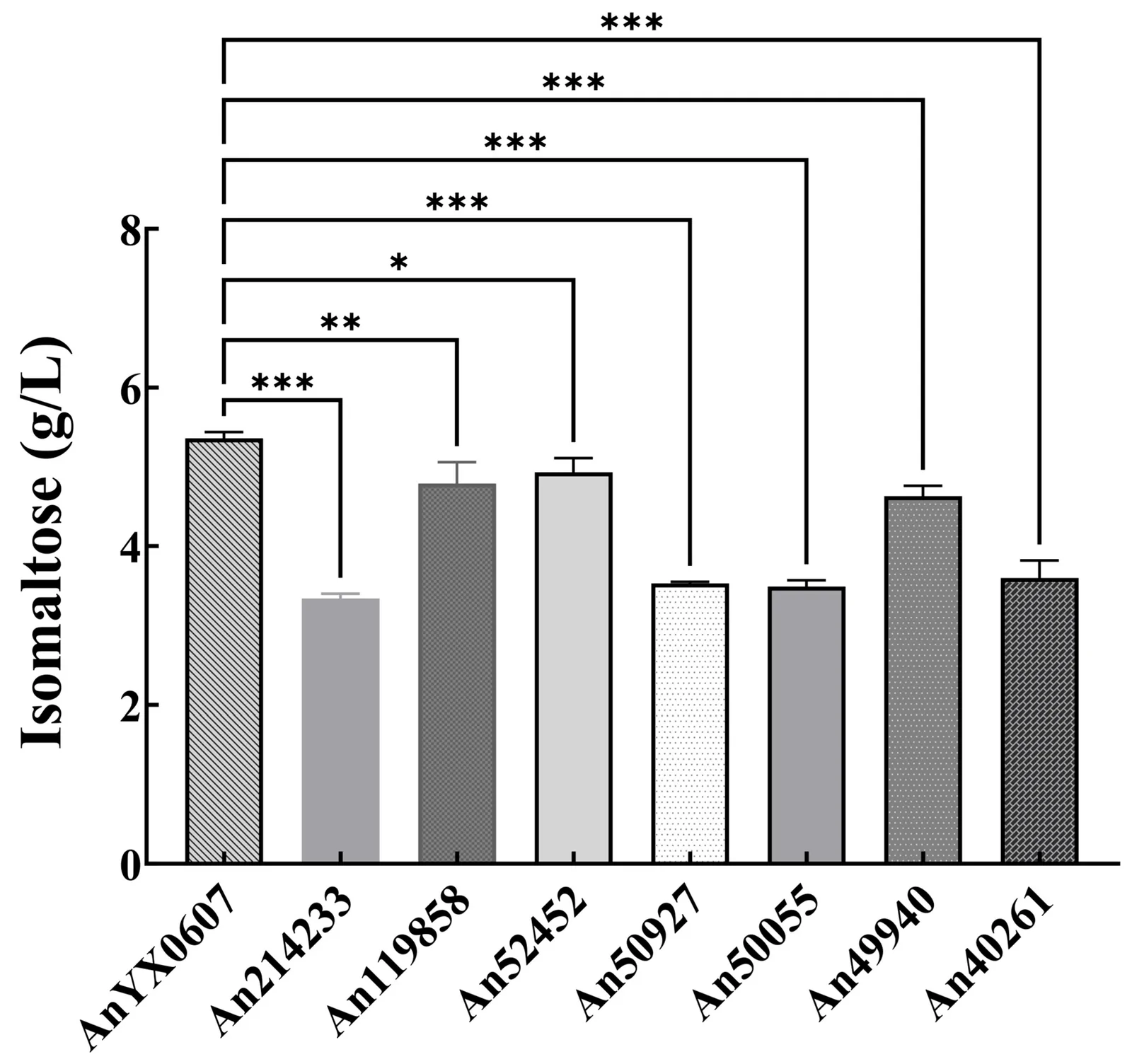
The isomaltose content of the AnYX0607 and gene-disrupted strains. Data are mean ± SD; *n* = 3; the * symbol represents *p* < 0.05; the ** symbol represents *p* < 0.01; and the *** represents *p* < 0.001 compared with AnYX0607.

The isomaltose concentrations in the fermentation broth of the α-glucosidase gene-disrupted strains and AnYX0607 after 96 h of fermentation were determined by HPLC, and the results are shown in Figure 10.

As shown in Figure 10, after 96 h of cultivation, the isomaltose content in the α-glucosidase gene-disrupted strains was significantly reduced compared with that in AnYX0607. Compared with AnYX0607, the isomaltose content of An214233, An119858, An52452, An50927, An50055, An49940, and An40261 decreased by 37.69%, 10.63%, 8.02%, 34.14%, 34.89%, 13.62%, and 32.84%, respectively, suggesting that these α-glucosidase genes are associated with isomaltose accumulation. Among them, the isomaltose content of An214233, An50927, An50055, and An40261 decreased by more than 30%, indicating that these genes may play a major role in the production of isomaltose.

### 3.8. Determination of Total Sugar and Reducing Sugar Content in Gene-Disrupted Strains and AnYX0607

**Figure 11:**
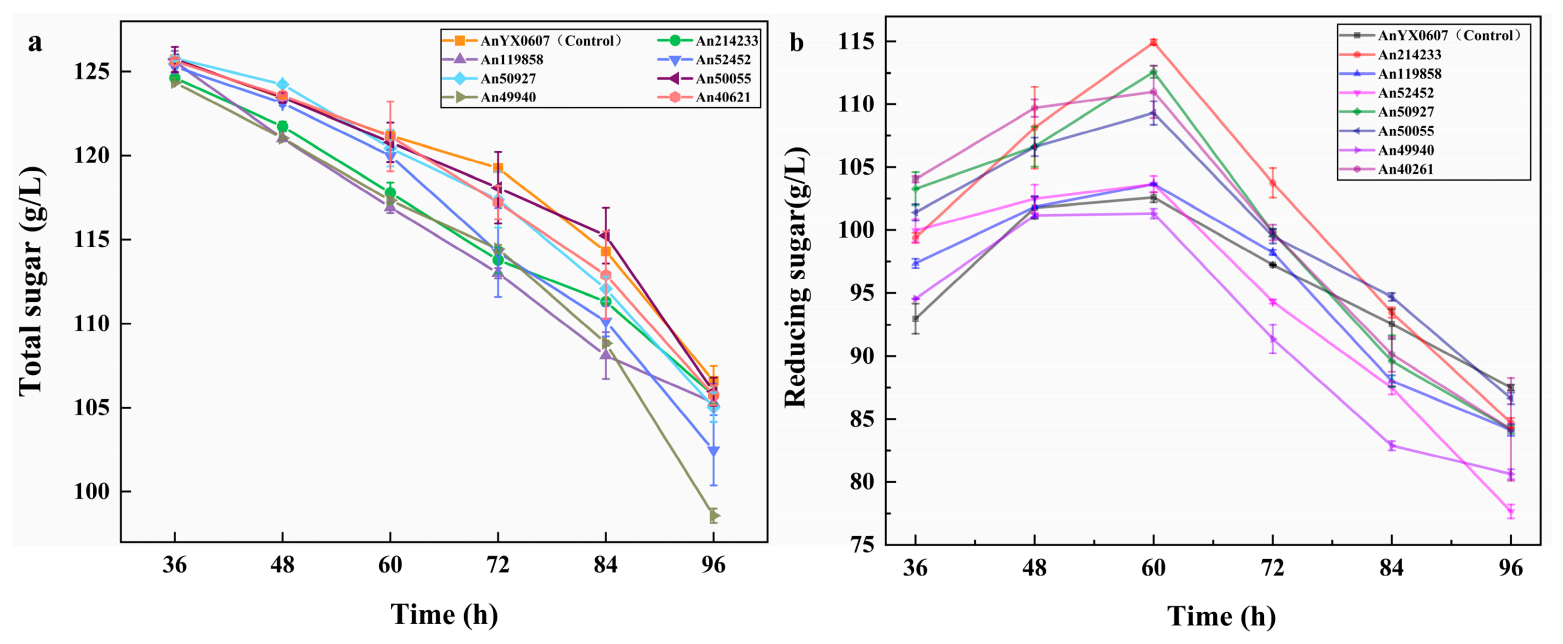
The content of total sugar and reducing sugar of AnYX0607 and gene-disrupted strains: (**a**) total sugar content of each strain at different times; (**b**) reducing sugar content of each strain at different times.

After 36 h of fermentation, samples were collected every 12 h, and the total sugar and reducing sugar contents in the fermentation broths of the gene-disrupted strains and AnYX0607 were determined. The results are shown in Figure 11.

As shown in Figure 11a, the total sugar content in the fermentation broth of all strains decreased progressively over time. At 96 h, compared with AnYX0607, the total sugar content of the gene-disrupted strains decreased by 0.79%, 1.22%, 3.88%, 1.47%, 0.60%, 7.53%, and 0.81%, respectively. Statistical analysis using the procedure described in Section 2.11 showed that the total sugar content at 96 h was significantly lower in An52452 and An49940 than in AnYX0607.

As shown in Figure 11b, during the fermentation period from 36 to 60 h, the reducing sugar content in the fermentation broth of all strains exhibited an upward trend. This upward trend during the early fermentation stage generally corresponds to the rapid vegetative growth phase, in which endogenous amylolytic enzymes, including glucoamylase and α-amylase, contribute to polysaccharide hydrolysis. Notably, all engineered strains in this study carry an integrated *glaA* overexpression cassette, which may also enhance extracellular starch hydrolysis and thereby interfere with the dynamic changes in reducing sugar levels. From 60 to 96 h, the reducing sugar content gradually decreased, indicating an increased rate of sugar utilization by the cells during this period, accompanied by the accumulation of citric acid. At 96 h, compared with AnYX0607, the reducing sugar content of the gene-disrupted strains decreased by 3.18%, 3.84%, 11.26%, 3.81%, 0.97%, 7.86%, and 3.78%, respectively. Statistical analysis using the procedure described in Section 2.11 showed that the reducing sugar content at 96 h was significantly lower in An52452 and An49940 than in AnYX0607.

In summary, after 96 h of fermentation, all gene-disrupted strains exhibited lower total and reducing sugar contents than the parental strain AnYX0607. However, the difference was small, mainly because the initial sugar concentration in the medium was relatively high and the strain did not fully utilize the sugar under shake-flask fermentation conditions. Among these gene-disrupted strains, An49940 exhibited the best overall performance, indicating that An49940 may be a promising target for improving carbon source utilization efficiency and reducing residual sugar levels.

### 3.9. Determination of Citric Acid Titer in Gene-Disrupted Strains and AnYX0607

**Figure 12:**
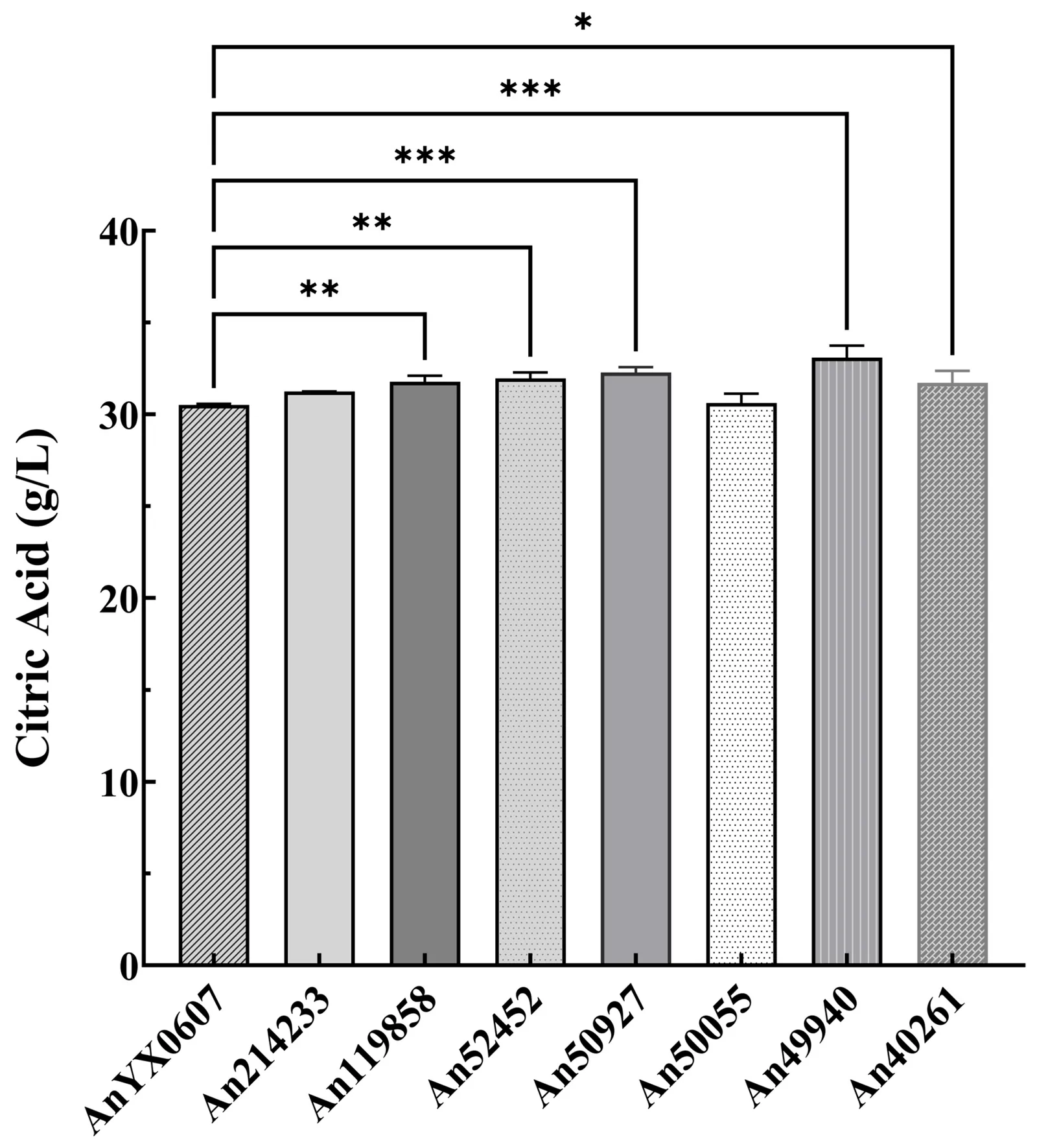
Citric acid titers of AnYX0607 and the gene-disrupted strains. Data are presented as mean ± standard deviation (SD) from three independent biological replicates (*n* = 3). Statistical analysis was performed using one-way ANOVA followed by Dunnett’s multiple-comparison test, with AnYX0607 as the reference strain. The * symbol represents *p* < 0.05; the ** symbol represents *p* < 0.01; *** represents *p* < 0.001 compared with AnYX0607.

The citric acid titers in the fermentation broth of the α-glucosidase gene-disrupted strains and AnYX0607 after 96 h of fermentation were determined by HPLC, and the results are shown in Figure 12. Compared with AnYX0607, citric acid titers increased by 2.39%, 4.16%, 4.72%, 5.80%, 0.36%, 8.46%, and 3.97% in An214233, An119858, An52452, An50927, An50055, An49940, and An40261, respectively. The increases observed in An119858, An52452, An50927, An49940, and An40261 were statistically significant. These results indicate that combining the disruption of α-glucosidase genes with *glaA* overexpression can improve citric acid production.

An49940 achieved the highest citric acid production among all gene-disrupted strains. It has been reported that high-titer citric acid strains exhibit compact hyphal pellets and thick hyphae [26,27,28]. Compared with the other strains, An49940 showed the most pronounced changes in pellet compactness and hyphal thickness and also exhibited the best performance in both total sugar and reducing sugar consumption. The combination of these phenotypic changes may have contributed to the higher citric acid production observed in An49940.

## 4. Discussion

Industrial production of citric acid typically employs a deep-tank liquid fermentation process using starch as the raw material [29]. After fermentation, 2–3% residual sugars remained in the fermentation broth, of which isomaltose accounted for 69.45%. The accumulation of such residual sugars not only represents a loss of fermentable carbon but also reduces the overall sugar utilization efficiency. Recent studies have also shown that enhancing cellular carbon uptake can improve citric acid production [30,31]. Therefore, reducing the generation of non-fermentable sugars and enhancing the availability of fermentable monosaccharides are effective strategies to increase citric acid titer.

Certain α-glucosidases can catalyze transglycosylation reactions during starch degradation, generating non-fermentable oligosaccharides such as isomaltose [13,14]. In addition, *agdB* from *A. niger* has been shown to exhibit transglucosylation activity toward oligosaccharide substrates [15]. Collectively, these findings imply that α-glucosidase-encoding genes participate in isomaltose biosynthesis. Disruption of these genes therefore represents an effective strategy to reduce the accumulation of non-fermentable sugars in the fermentation system and improve sugar utilization efficiency. The results of this study showed that the isomaltose content in the fermentation broth of all α-glucosidase gene disruption strains was significantly reduced, indicating that these genes are involved in isomaltose synthesis. Notably, genes 214233, 50055, 50927, 49940, and 40261 showed highly significant effects, suggesting that these genes make important contributions to isomaltose formation.

In the citric acid fermentation system, hydrolysis of starchy raw materials releases abundant utilizable monosaccharides, providing substrates for cell growth and metabolism, which helps improve sugar utilization efficiency and reduce residual sugar content in the fermentation broth. Based on this, the *glaA* gene expression cassette was integrated into the α-glucosidase gene loci for simultaneous overexpression of GlaA with disruption of seven α-glucosidase genes. However, since the *glaA* expression cassette was integrated at different gene loci, *glaA* expression may differ among the gene-disrupted strains. Therefore, the observed fermentation phenotypes should be interpreted in the context of both α-glucosidase gene disruption and *glaA* overexpression. These results suggest that α-glucosidase gene disruption combined with *glaA* overexpression reduces residual sugar accumulation and increases citric acid titers.

In this study, we found that the mycelial morphology of all α-glucosidase gene disruption strains was altered, manifested as a decrease in mycelial pellet diameter and an increase in hyphal diameter. Previous studies have demonstrated that overexpression of the *glaA* gene does not alter the mycelial morphology of the strain [32], indicating that the morphological changes were caused by the disruption of the α-glucosidase genes. Some studies also showed that smaller pellets facilitate nutrient and oxygen transfer during fermentation, while thicker hyphae may enhance metabolic activity and product accumulation capacity [26]. Smaller pellets can increase the surface-to-volume ratio and shorten diffusion distances, thereby alleviating mass-transfer limitations, whereas thicker hyphae may provide more intracellular space and metabolic capacity for product synthesis [33]. Consistent with this interpretation, morphology-controlled seed preparation has been reported to improve citric acid production during semi-continuous cultivation of *A. niger* [28]. A recent study further showed that repressing *flbE* reduced pellet size and increased citric acid production; these phenotypic changes were accompanied by transcriptional changes in genes involved in cell-wall remodeling, glucose uptake, citrate export, and extracellular hydrolase production [27]. These findings support a link between fungal morphology and metabolism.

Among all disruption strains, An49940 showed the most pronounced change in citric acid titer. Its smaller pellets and thicker hyphae may have improved oxygen and nutrient transfer and enhanced metabolic capacity; meanwhile, its markedly reduced isomaltose content indicated decreased accumulation of non-fermentable sugars and more carbon available for citric acid synthesis. These effects may have jointly contributed to the superior performance of An49940, with the lowest residual sugar level and the highest citric acid titer. This interpretation is consistent with recent studies showing that citric acid production is influenced by broader carbon metabolism. Modulating *gsdA* altered glucose-specific yield, mycelial growth, and final citric acid titer, indicating that carbon redistribution may involve a trade-off between product yield and biomass formation [34]. Likewise, a citrate-synthase overexpression study highlighted that improving citric acid production requires coordinated regulation of the broader metabolic network rather than manipulation of a single biosynthetic step [35]. Systems metabolic engineering studies have similarly emphasized that carbon utilization, precursor supply, membrane transport, and byproduct formation are interconnected determinants of citric acid production [8,9]. Accordingly, the high citric acid titer of An49940 likely resulted from the combined effects of improved sugar utilization, altered morphology, and changes in carbon metabolism.

Nevertheless, the internal regulatory relationships between α-glucosidase gene disruption and mycelial morphology, oxygen transfer efficiency, carbon flux redistribution, and citric acid anabolism still require further in-depth investigation. Future studies could employ transcriptomic and metabolomic profiling to elucidate the molecular regulatory network downstream of key α-glucosidase genes and to further validate the contributions of these morphological and metabolic changes under industrial fermentation conditions.

## 5. Conclusions

In this study, seven individual α-glucosidase gene-disrupted strains were constructed, and the roles of these genes in isomaltose formation, sugar utilization, mycelial morphology, and citric acid production were investigated. All seven α-glucosidase genes were involved in isomaltose formation, among which genes 214233, 50927, 50055, 49940, and 40261 played dominant roles. Since the *glaA* expression cassette was integrated at different genomic loci, the observed phenotypes likely reflect the combined effects of α-glucosidase gene disruption and strain-dependent *glaA* expression. Accordingly, disruption of α-glucosidase genes combined with *glaA* overexpression effectively reduced residual sugar accumulation and promoted citric acid production. These findings demonstrate that combining α-glucosidase gene disruption with *glaA* overexpression is an effective strategy to reduce non-fermentable sugar accumulation and enhance citric acid fermentation efficiency in *Aspergillus niger*.

## Figures and Tables

**Table 1:** Sugar composition analysis of citric acid-fermented liquid.

Fermented Liquid Ingredients	Peak Area	Total Area	Area Percentage (%)
Isomaltose	179,821,773	258,933,924	69.45
Alpha-D-Glucopyranoside	59,569,685		23.01
1,6-Anhydro-β-d-glucose	12,808,021		4.94
Arabinofuranose	502,911		0.19
Alpha-D-Galactofuranose	3,069,673		1.19
Mannose	30,686		0.01
D-Xylose	2,492,168		0.96
2-O-Glycerol-alpha-D-galactopyranoside	639,007		0.25

**Table 2:** Comparison of α-glucosidase sequences from *A. niger* AnYX0607 with ATCC 1015.

Gene ID	Gene Annotation	Nucleotide Similarity	AA Similarity (%)
214233	*α*-glucosidase	99.26%	100%
119858	*α*-glucosidase	99.81%	99.88%
52452	putative *α*-glucosidase	99.89%	99.83%
50927	putative *α*-glucosidase	100%	100%
50055	putative *α*-glucosidase	100%	100%
49940	putative *α*-glucosidase	98.86%	99.41%
40261	putative *α*-glucosidase	99.29%	99.33%

## Data Availability

The data are available from the corresponding authors upon request.

## Supplementary Materials

The following supporting information can be downloaded at https://www.mdpi.com/article/10.3390/jof12100764/s1, Table S1: The strains and plasmids used in this study; Table S2: The primers used in this study; Figure S1: Representative HPLC chromatograms used for citric acid determination.
