# Supplementary material for: Decreasing the Non-Fermentable Sugar Concentration in Aspergillus niger by Destroying the α-Glucosidase Genes
Source: J Fungi (Basel). 2026 Oct 11;12(10):764. doi: 10.3390/jof12100764 (PMC13705924; doi:10.3390/jof12100764)
Supplement: Supplementary file 1 [file jof-12-00764-s001.zip › jof-4518979-supplementary.pdf]

Supplementary Materials

Table S1. The strains and plasmids used in this study

Strain or Plasmid	Genotype or Relevant genotype/description	Source or reference
AnYX0607		Our lab
An214233	△*214233*::*glaA*	This study
An119858	△*119858*::*glaA*	This study
An52452	△*52452*::*glaA*	This study
An50927	△*50927*::*glaA*	This study
An50055	△*50055*::*glaA*	This study
An49940	△*49940*::*glaA*	This study
An40261	△*40261*::*glaA*	This study
pFC332	Amp^r	Purchased from Addgene
pUC57-sgRNA	Amp^r	Our lab
pUC57-*glaA*	Amp^r, containing the *glaA* overexpression cassette	Our lab
pFC332-target-214233-sgRNA	Hyg^r, Amp^r, α-glucosidase gene disruption plasmid	This study
pFC332-target-119858-sgRNA	Hyg^r, Amp^r, α-glucosidase gene disruption plasmid	This study
pFC332-target-50927-sgRNA	Hyg^r, Amp^r, α-glucosidase gene disruption plasmid	This study
pFC332-target-50055-sgRNA	Hyg^r, Amp^r, α-glucosidase gene disruption plasmid	This study
pFC332-target-49940-sgRNA	Hyg^r, Amp^r, α-glucosidase gene disruption plasmid	This study
pFC332-target-40261-sgRNA	Hyg^r, Amp^r, α-glucosidase gene disruption plasmid	This study
pFC332-target-52452-sgRNA	Hyg^r, Amp^r, α-glucosidase gene disruption plasmid	This study
pUC57-214233-LB-glaA-214233-RB	Amp^r, containing the *glaA* overexpression cassette	This study
pUC57-119858-LB-glaA-119858-RB	Amp^r, containing the *glaA* overexpression cassette	This study
pUC57-50927-LB-glaA-50927-RB	Amp^r, containing the *glaA* overexpression cassette	This study
pUC57-50055-LB-glaA-50055-RB	Amp^r, containing the *glaA* overexpression cassette	This study
pUC57-49940-LB-glaA-49940-RB	Amp^r, containing the *glaA* overexpression cassette	This study
pUC57-40261-LB-glaA-40261-RB	Amp^r, containing the *glaA* overexpression cassette	This study
pUC57-52452-LB-glaA-52452-RB	Amp^r, containing the *glaA* overexpression cassette	This study

Table S2. The primers used in this study

Primers	NCBI locus tag	GenBank accession number	Sequence (5′ → 3′)	Primer source
214233-F	ASPNIDRAFT_214233	EHA20839.1	CATGGTGAAGTTGACGC ATCTCCTTGCC	Designed independently in this study
214233-R	ASPNIDRAFT_214233	EHA20839.1	TACCATTCCAATACCCA GTTTTCCGCCCAT	Designed independently in this study
119858-F	ASPNIDRAFT_119858	EHA26885.1	ATGTTGGGGTCTTTGCT TTTACTCTTACC	Designed independently in this study
119858-R	ASPNIDRAFT_119858	EHA26885.1	GTCCGTGCTGACTTCCC CAGCAAGG	Designed independently in this study
52452-F	ASPNIDRAFT_52452	EHA23512.1	ATGGCCAAATCCGCCTC CCAGATTCAC	Designed independently in this study
52452-R	ASPNIDRAFT_52452	EHA23512.1	TTAGGCCTCCACCAAAA GAACAACTGC	Designed independently in this study
50927-F	ASPNIDRAFT_50927	EHA27488.1	ATGTGTCACAAGTCCAA TTACTCATCCCCGAA	Designed independently in this study
50927-R	ASPNIDRAFT_50927	EHA27488.1	TTAACTCAAGGTTCTTT TCTTTGGAAGGCTCCA	Designed independently in this study
50055-F	ASPNIDRAFT_50055	EHA28539.1	ATGTCCAACCGTTGGAC CCTACTGC	Designed independently in this study
50055-R	ASPNIDRAFT_50055	EHA28539.1	CTAAAACTCAATCCGCC ATGTCTTTCCAATGCC	Designed independently in this study
49940-F	ASPNIDRAFT_49940	EHA19157.1	ATGCCTCAAAAAGAATT CGTCCCCAAGACTT	Designed independently in this study
49940-R	ASPNIDRAFT_49940	EHA19157.1	CTAGTTCTTCAAGGTAT AAACCACCCGTCCCCGG	Designed independently in this study
40261-F	ASPNIDRAFT_40261	EHA23680.1	ATGGCGCCTCCTCCGTC CTCTTAC	Designed independently in this study
40261-R	ASPNIDRAFT_40261	EHA23680.1	TCACTGGTTCTTCTTCTC CGTCTCCTC	Designed independently in this study
214233-LB-Xho I-F	ASPNIDRAFT_214233 (homologous arm)	EHA20839.1	AATCTCGAGCCAAAGG GGGTATCAC	Designed independently in this study
214233-LB-Bgl II-R	ASPNIDRAFT_214233 (homologous arm)	EHA20839.1	AATAGATCTCCTGGCAG CCAGACGATCA	Designed independently in this study
214233-RB-BamH I-F	ASPNIDRAFT_214233 (homologous arm)	EHA20839.1	TATGGATCCCCACGGCA TTCCTGCATGACT	Designed independently in this study
214233-RB-Hind III-R	ASPNIDRAFT_214233 (homologous arm)	EHA20839.1	CTCAAGCTTATGCTGTG ACTAACCCGAGCG	Designed independently in this study
119858-LB-Xho I-F	ASPNIDRAFT_119858 (homologous arm)	EHA26885.1	GTTCTCGAGGTTGCATC CGACGCTTTGT	Designed independently in this study
119858-LB-Bgl II-R	ASPNIDRAFT_119858 (homologous arm)	EHA26885.1	CCAGGATCCATTACAGC AAGCCGTGACTTCA	Designed independently in this study

Primers	NCBI locus tag	GenBank accession number	Sequence (5′ → 3′)	Primer source
119858-RB-BamH I-F	ASPNIDRAFT_119858 (homologous arm)	EHA26885.1	TTCGGATCCTAAGCATAAACTCTGCCGCAGC	Designed independently in this study
119858-RB-Hind III-R	ASPNIDRAFT_119858 (homologous arm)	EHA26885.1	CGTAAGCTTATCATCATCCGTGTTCTCCTCTGC	Designed independently in this study
52452-LB-Xho I-F	ASPNIDRAFT_52452 (homologous arm)	EHA23512.1	TTGCTCGAGGCGTTACATCCATCAGCTTGGC	Designed independently in this study
52452-LB-Bgl II-R	ASPNIDRAFT_52452 (homologous arm)	EHA23512.1	GGAAGATCTAGGTCAGAGGGGGCACAAG	Designed independently in this study
52452-RB-BamH I-F	ASPNIDRAFT_52452 (homologous arm)	EHA23512.1	TGCGGATCCACAGTGGAGTCAAGTATGTTTTTCGTAGT	Designed independently in this study
52452-RB-Hind III-R	ASPNIDRAFT_52452 (homologous arm)	EHA23512.1	GCGAAGCTTCATTTTGGATATAGATGGTAGGGTAG	Designed independently in this study
50927-LB-Xho I-F	ASPNIDRAFT_50927 (homologous arm)	EHA27488.1	AAACTCGAGGCATGGCGAACGCCCACA	Designed independently in this study
50927-LB-Bgl II-R	ASPNIDRAFT_50927 (homologous arm)	EHA27488.1	CGGAGATCTTCAGGCAGTTCCAATCTTCACTGA	Designed independently in this study
50927-RB-BamH I-F	ASPNIDRAFT_50927 (homologous arm)	EHA27488.1	GTGGGATCCCAGGTTGCGTGGTATCACTACTAGTAC	Designed independently in this study
50927-RB-Hind III-R	ASPNIDRAFT_50927 (homologous arm)	EHA27488.1	CCGAAGCTTAACGTCGTTTCAACAGAACCCATCT	Designed independently in this study
50055-LB-Xho I-F	ASPNIDRAFT_50055 (homologous arm)	EHA28539.1	ATTCTCGAGAGCAGAGCTTCAGAGCAACG	Designed independently in this study
50055-LB-Bgl II-R	ASPNIDRAFT_50055 (homologous arm)	EHA28539.1	GCGAGATCTACACTCAACCTCTGAAAGAAGAACTATCA	Designed independently in this study
50055-RB-BamH I-F	ASPNIDRAFT_50055 (homologous arm)	EHA28539.1	AAGGGATCCCACGAGGATATGGATTGAGCACCC	Designed independently in this study
50055-RB-Hind III-R	ASPNIDRAFT_50055 (homologous arm)	EHA28539.1	ACCAAGCTTTCAACAAGACTACCTGCACCCC	Designed independently in this study
49940-LB-Xho I-F	ASPNIDRAFT_49940 (homologous arm)	EHA19157.1	ATGCTCGAGCGGGATAACATGACGTGGAAGTGG	Designed independently in this study
49940-LB-Bgl II-R	ASPNIDRAFT_49940 (homologous arm)	EHA19157.1	GGTAGATCTTCACACTGGGGTATGGAGGGA	Designed independently in this study
49940-RB-BamH I-F	ASPNIDRAFT_49940 (homologous arm)	EHA19157.1	GGCGGATCCGGATATTGGAAAGACTGTGGAGTATAAGGG	Designed independently in this study
49940-RB-	ASPNIDRAFT_49940	EHA19157.1	GGCAAGCTTCAAGGAT	Designed independently

Primers	NCBI locus tag	GenBank accession number	Sequence (5′ → 3′)	Primer source
Hind III-R	(homologous arm)		GACGAATAGAGTCGAA GTGTTG	in this study
40261-LB-Xho I-F	ASPNIDRAFT_40261 (homologous arm)	EHA23680.1	GTACTCGAGATGGCCTG ATCAGGG	Designed independently in this study
40261-LB-Bgl II-R	ASPNIDRAFT_40261 (homologous arm)	EHA23680.1	GGAAGATCTCTTGGGCG AGAGTTTTGAAATCCTG	Designed independently in this study
40261-RB-BamH I-F	ASPNIDRAFT_40261 (homologous arm)	EHA23680.1	GGCGGATCCTGTTGCTT CTCATGCACTGTATACT AAGAGT	Designed independently in this study
40261-RB-Hind III-R	ASPNIDRAFT_40261 (homologous arm)	EHA23680.1	GGGAAGCTTCATCCGCC CCAATGTTTGCTA	Designed independently in this study
glaA-F	ASPNIDRAFT_213597	EHA21384.1	CAACTGGGGATCGGTA CCATGGCGTCCCTC	Designed independently in this study
glaA-R	ASPNIDRAFT_213597	EHA21384.1	GTCAGTCACCCTCTAGA TCACTGCCAACTATCGT CA	Designed independently in this study

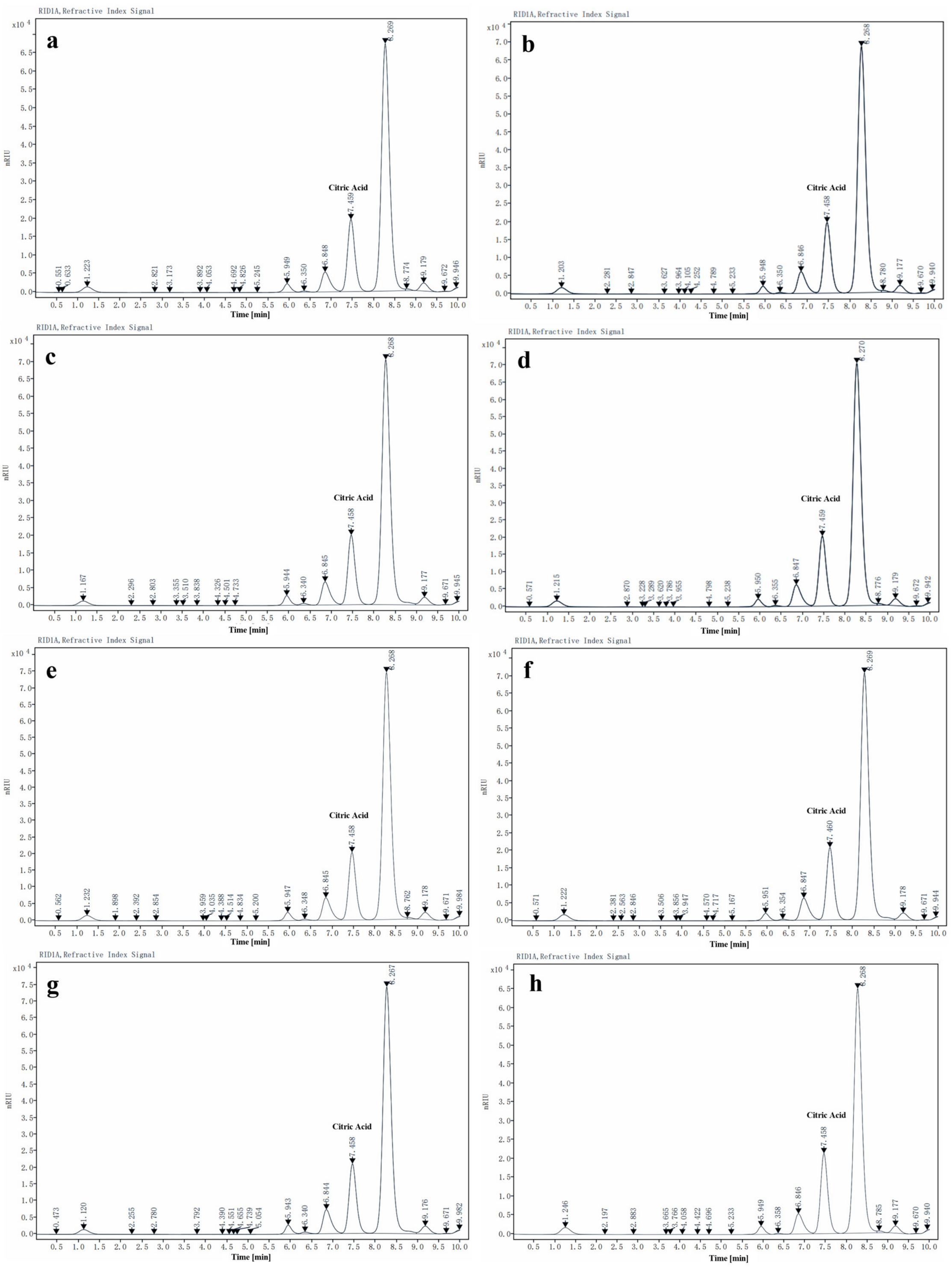

Figure S1. HPLC chromatograms of fermentation broth samples used for the determination of citric acid: (a) AnYX0607; (b) An214233; (c) An119858; (d) An52452; (e) An50927; (f) An50055; (g) An49940; and (h) An40261. The peak around 7.46 min was identified as citric acid by comparing it with the citric acid standard. The chromatographic signals were monitored using a refractive index detector (RID) under the HPLC conditions described in Section 2.10 of the revised manuscript.
